# Association of fourteen years diet quality trajectories and type 2 diabetes mellitus with related biomarkers

**DOI:** 10.18632/aging.202771

**Published:** 2021-03-26

**Authors:** Therese Martin Cheteu Wabo, Victorine Raïssa Ngo Nkondjock, Justina Ucheojor Onwuka, Changhao Sun, Tianshu Han, Jackson Sira

**Affiliations:** 1 Department of Nutrition and Food Hygiene, Harbin Medical University, Harbin 150081, China; 2 Department of Biomedical Sciences, Faculty of Sciences, University of Ngaoundere, Ngaoundere, Cameroon; 3 Department of Epidemiology, Public Health College, Harbin Medical University, Harbin 150081, China; 4 Center for Endemic Disease Control, Chinese Center for Disease Control and Prevention, Harbin Medical University, Harbin 150081, China

**Keywords:** diet, quality, trajectories, T2DM, biomarkers

## Abstract

Diet quality fluctuates throughout one’s adulthood, yet it remains unclear how long-term diet quality changes are related to type 2 diabetes mellitus (T2DM) and its biomarkers. We aimed to examine the association of long-term diet quality with T2DM and its biomarkers. Diet quality was assessed by the revised DBI-07, in which diet quality distance levels (DQD) represented the overall diet quality. Participants were clustered into classes sharing similar DQD levels using latent class mixed model. We used Cox regression and random effect linear regression to assess DQD trajectories’ association with T2DM and its biomarkers. Three DQD trajectories were derived: moderate to gradual decrease, high to moderate, high stable DQD level representing 2.14%, 6.18% and, 91.68% of the population. Compared to class 1, class 2 and 3 were associated with an increased risk of T2DM [HR=4.40; 95%CI: 2.02-9.59]; [HR=3.68; 95% CI: 2.11-6.43]. When class 3 was used as a reference, class 1 was also associated with an increased risk of TDM [HR=2.71; 95%CI: 1.55-4.73]. Our findings suggest that a long-term unhealthy diet is associated with an increased risk of T2DM. Gradually reducing DQD level may not make a difference, which establishes the importance of promoting healthy eating habits at early adulthood.

## INTRODUCTION

Type 2 diabetes mellitus (T2DM) is a major contributor to the global burden of disease and represents one of the ten greatest risk factors for all-cause mortality worldwide [1, 2]. In 2017, 425 million people were estimated to have diabetes, and it is expected to increase to 592 million by 2035 [1–3]. In China, the prevalence of T2DM has increased from 1.6% in the 1980s to 5.5% in 2001, 9.7% in 2008, and 11.6% in 2010. [4–7] Recently, the estimated number of individuals with T2DM has reached 110 million in China, making it the country with the largest number of individuals suffering from T2DM in the world [8]. In addition to the complex interactions between human behavior, environmental and genetic predispositions, it is established that diet is a key factor determining T2DM [9]. In recent years, China has experienced rapid, profound socio-economic progress, social changes, diseases patterns modifications, and a profound nutritional transition [8, 10, 11], shifting from traditional diet to western diet [12, 13]. In view of these profound changes in diet, many studies have linked the overall diet quality to T2DM in China using Diet quality indices.

Diet quality indices are implements used to evaluate individuals’ overall diet quality, examine the diet-disease relationships in a population, and set recommendations for good health outcomes [14, 15]. Several studies have assessed Chinese adults' diet quality and reported an imbalanced diet quality with diet patterns. They were characterized by low intake of fruits, vegetables, legumes, milk, nuts, and high intake of meat products, salt, alcohol, which has been associated with T2DM and other chronic diseases [16–20]. However, these studies have focused on the significant role of the overall diet quality in predicting T2DM, including diet quality from only one point in time [14, 15, 21, 22]. Assessing the overall diet quality at baseline implies ignoring the variation of food consumption, and consequently, may not capture the long-term cumulative effects of diet quality changes on T2DM. Since diet quality fluctuates and is unsteady throughout one’s adulthood, it remains unclear how long-term diet quality changes are related to T2DM and its biomarkers. Hence, in this study, we used repeated dietary data for Chinese adults between 1997-2011 to examine the association of long-term changes in diet quality scores as assessed by the revised DBI-07 (Diet Balanced Index-2007) with T2DM and its biomarkers. To represent the long-term diet quality changes, we used latent class mixed models (LCMM) to identify participants with similar diet quality scores and cluster them into classes. The identified classes were used as an exposure to evaluate the future risk of T2DM and its biomarkers among Chinese adults.

## RESULTS

### Diet quality distance trajectories

**Figure 1:**
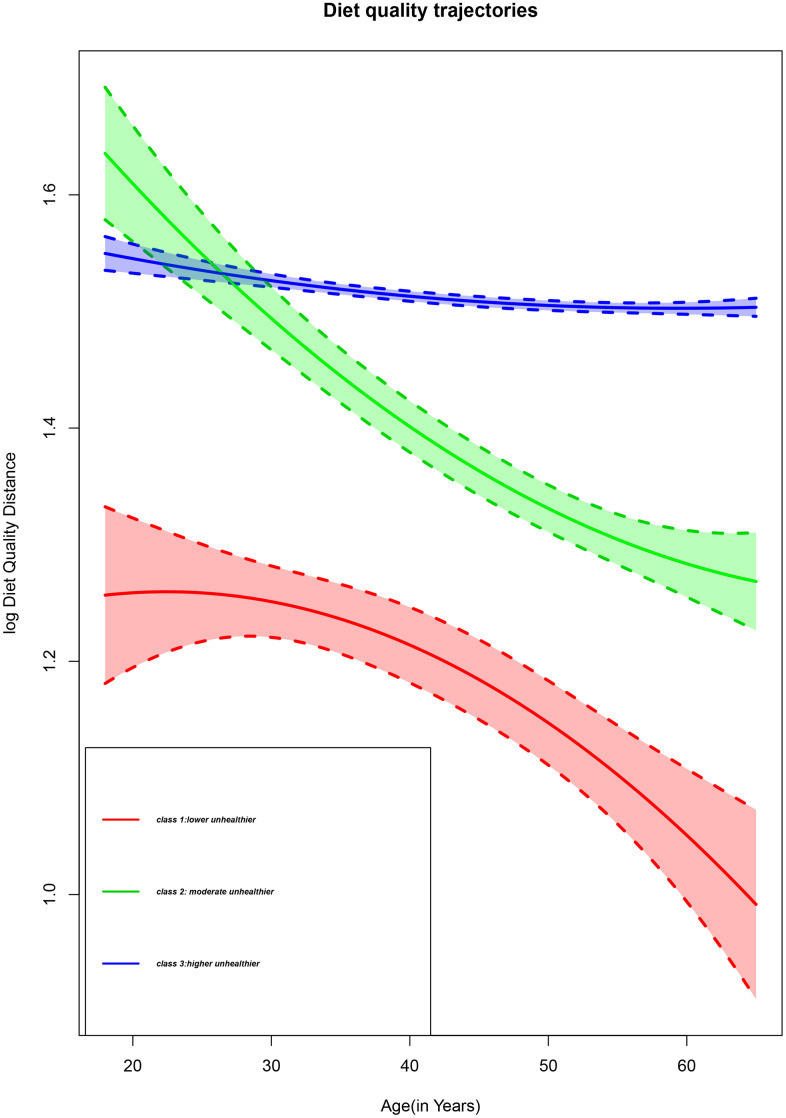
**Diet quality trajectories of Chinese adults from the CHNS 1997-2011.**

We identified three trajectories with comparable DQD throughout 14 years of diet intake, as shown in Figure 1. The first trajectory labelled class 1 represented 2.14 % of the participants with a moderate to persistent decline of DQD level corresponding to Chinese adults who decreased their DQD level from moderate to low. The second trajectory identified as class 2 represented 6.18% of the participants with a high to persistently moderate DQD level, which corresponds to Chinese adults with a high DQD level at early adulthood whose level persistently declined and remained moderate with age. The third trajectory, named class 3, represented 91.68% of the participants with a persistently high trajectory for those with a high DQD level throughout adulthood. According to the elevated DQD level found in all trajectories, we defined the three classes as lower unhealthier group, moderate unhealthier group, and higher unhealthier group for class 1, class 2, and class 3.

### Participants’ characteristics by trajectory class

Participants within the three DQD trajectories class had distinct clinical profiles, as shown in Table 1. Significant differences in sex, age, DBP among the three classes were observed. The proportion of men was the largest in class 2 (68.8%), and women were more represented in class 3 (53.6%). Participants in class 1 were younger (mean (SD) = 38.48 (10.78)), while participants in classes 2 and 3 were relatively older (mean (SD) = 48.02 (8.51) and (mean (SD) = 45.01(11.32)) years respectively. The DBP significantly varied across different trajectories. There was no significant difference in BMI, PAL, SBP, hypertension status, smoking status across DQD trajectories (*p-value > 0.05).* Significant distinct demographic profiles were also observed across trajectories. The participants in the first class tended to have higher education (10.7%), with a high urbanicity index (56.0%), lived in the northern region (60.9%), all *p-value* < 0.05. Participants of all classes tended to live in rural areas, with the highest percentage of 67.5% represented in the third class.

**Table 1:** Participants’ characteristics by trajectory class.

**Baseline variables**	**Trajectories**			***p-value***
	**Class1 (n=173)**	**Class 2 (n=500)**	**Class3(n=7415)**	
Age (years)	38.48 (10.78)	48.02 (8.51)	45.01(11.32)	<0.001
Gender (%)				<0.001
Male	65.5	68.8	46.4	
Female	34.5	31.1	53.6	
Income	2387.30 (3998.56)	3213.82 (6325.49)	5002.58 (10422.08)	<0.001
BMI (kg/m^2^)	22.11 (3.91)	22.13 (3.59)	22.00 (3.80)	0.277
PAL(MET-h/week)	268.86 (219.34)	268.29 (218.76)	265.05 (211.67)	0.741
SBP (mmHg)	115.81 (16.03)	116.87 (16.64)	116.03 (16.56)	0.095
DBP (mmHg)	76.26 (10.59)	76.44 (10.85	75.61 (10.74)	0.002
Urban Index	67.72 (19.53)	61.65 (21.29)	60.60 (20.33)	<0.001
Energy (Kcal)	1944.50 (609.45)	2040.23 (642.88)	2056.25 (634.99)	<0.001
Regions (%)				<0.001
North	60.9	51.5	26.4	
Central	22.8	27.7	36.2	
East	16.3	20.9	37.4	
Area (%)				<0.001
Rural	54.4	58.4	67.5	
Urban	45.6	41.6	32.5	
Urbanicity (%)				<0.001
Low	17.8	30.6	30.2	
Medium	26.2	26.8	31.6	
High	56.0	42.6	38.3	
Smoking status (%)				0.318
Yes	91.2	87.6	89.5	
No	8.8	12.4	10.5	
Education level (%)				<0.001
Illiteracy	14.9	21.8	23.3	
Low	51.1	51.5	53.2	
Medium	23.3	18.6	18.4	
High	10.7	8.2	5.1	
Hypertension (%)				0.09
Yes	10.9	14.2	12.9	
No	89.1	85.8	87.1	

Continuous variables are expressed as mean (SD); and categorical variables as percentage (%).

### Participants’ food components scores frequency

Table 2 shows the scores for the DBI-07 food subgroups and the participants’ percentages with each score. Excessive (DBI-HBS) and insufficient (DBI-LBS) food intake were coexistent. In total, 43.6% and 32.6% of the participants consumed red meat products, poultry, and game in insufficient and excessive amounts, respectively. Cooking oil, alcohol, and salt were consumed in suitable quantities; almost 100% of the participants obtained a score of 0. Overall, 62% of the participants had high cereals intake, and 13.7% had reached the maximum score of +12. Fruits, vegetables, dairy, diet variety, drinking water, soybean, eggs, fish, and shrimp were consumed in small quantities by the majority. Almost 40% had a score between -8 and -7 for water intake, 47.4% of adults had a score between -6 and -5, representing the lower scores for soybean intake, 96.7%, 80.3%, and 75.1% of the participants had the lower score for dairy, fruits, fish, and shrimps’ intake respectively. Only 1.4%, 0.2%, 2%, and 0% had a score of 0 for water, dairy, fruits, and diet variety, respectively.

**Table 2:** Score for the DBI-07 food subgroups and percentages of the participants with each score.

**Score**	**Cereals**	**Vegetables**	**fruits**	**Dairy**	**soybean**	**Red meat products poultry and game**	**Fish and shrimp**	**Eggs**	**Cooking oil**	**Salt**	**Alcohol**	**Diet variety**	**Drinking water**
(-12) - (-11)	13											18.8	1.9
(-10) - (-9)	2.9											9.6	22.1
(-8) - (-7)	4.4											29	39.9
(-6) - (-5)	0.8	0.3	80.3	96.7	47.4							30.4	23.4
(-4) - (-3)	1.5	35.3	12.5	1.4	8.8	23.8	75.1	43.9				11	8.6
(-2) - (-1)	3.2	28.1	5.2	1.6	8.9	19.8	8.8	19				1.2	2.7
0-1	7.2	36.3	2	0.2	35.0	23.7	16.1	19.3	99.8	99.8	97.8	0	1.4
2-3	10.8					14.8		7.3	0.1	0.1	1.0		
4-5	12.5					17.8		10.5	0	0.1	1.2		
6-7	12.7												
8-9	10.2												
10-11	7.1												
12	13.7												

### Participants' food subgroups intake by DQD trajectory class

We observed significant differences in most food subgroups intake across all trajectories, as shown in Table 3. Participants of all trajectories had a significantly different intake of almost all food subgroups. Diet patterns across all classes tended to be low in alcohol, salt, cooking oil, fruits, dairy, diet variety, water, with sufficient vegetable intake. Cereals were consumed in appropriate amounts in class 3 (mean (SD) = 433.35g (290.30)), while class 1 and class 2 had higher cereals’ intake. Participants across class 3 had the lowest intake of meat products (mean (SD) = 38.19g (66.01)), alcohol (mean (SD) = 0.6g (2.8)), fish (mean (SD) = 23.11g (48.04)), fruits (mean (SD) =29.97g (96.22)), eggs(mean (SD) = 20.65g(32.46)), dairy (mean (SD) = 5.01g(30.49)), diet variety(mean (SD) = 59.08g (36.73)) and water(mean (SD) = 539.42 L (215.71)). Although they were all consumed in insufficient amounts, class 1 had higher amount of fruits (mean (SD) = 133.86g (173.08)), dairy(mean (SD) = 60.83g (104.03)), fish and shrimp(mean (SD) = 58.89g (76.92)), diet variety(mean (SD) = 92.79g (41.66)), and water(mean (SD) = 873.86L (592.12)). Red meat products, poultry and game intake were high in class 1(mean (SD) = 154.46g (126.38)). Participants in the second trajectory had an adequate intake of red meat products, poultry and game (mean (SD) = 71.19g (86.01)). Vegetables were consumed in adequate amounts in all trajectories. In terms of scores, the diet patterns mentioned above were somewhat confirmed in Supplementary Table 1, considering the participant’s distribution by food components score across all trajectories. Class 3 had the higher proportion of participants with a smaller negative score of (-6) to (-5) for fruits (81.1%), dairy (97.8%), soybean (48.3%), and the score of (-4) to (-3) for fish, shrimps (75.8%) and eggs (45.5%). Diet variety and water intake were inadequate for most participants across all trajectories. More than 40% of the participants had a low score of (-8) to (-7) for water intake and 30.6% for diet variety intake across class 3. Participants across class 3 had adequate intake for cereals’; 17.2% had reached the minimum score of 0. Class 1 had a higher percentage (44.1%) of the participants with a score of 0 for vegetable intake. Participants across class 1 had the smallest percentage of adults with the lower score of (-6) to (-5) for fruits (42.7%), dairy (73.1%), soybean (21.2%), and the score of (-4) to (-3) for fish and shrimps (30.6%), and eggs (6.3%). The same trajectory had a higher proportion of individuals with high meat products intake for an overall 56.3%, with almost 30% of the participants having a higher score of 4 for red meat products, poultry, and game intake. Throughout the third trajectory, only 7.5% of the participants had a high score of 4 for meat products intake.

**Table 3:** DBI-07 food subgroups by DQD trajectories.

**Food subgroups**	**Trajectories**			***p-value***
	**Class1 (n=173)**	**Class2(n=500)**	**Class3(n=7415)**	
Cereals (g/day)	523.26(295.88)	535.62(337.90)	433.35(290.30)	<0.001
Vegetable (g/day)	496.43(235.38)	472.27(238.71)	373.98(190.49)	<0.001
Fruits(g/day)	133.86(173.08)	83.88 (177.33)	29.97 (96.22)	<0.001
Dairy (g/day)	60.83 (104.03)	26.96 (85.08)	5.01 (30.49)	<0.001
Soybean (g/day)	80.09(113.21)	65.33 (90.44)	33.52 (56.50)	<0.001
Red meat products poultry and game(g/day)	154.46(126.38)	71.19 (86.01)	38.19 (66.01)	<0.001
Fish and shrimp(g/day)	58.89 (76.92)	48.41 (76.69)	23.11 (48.04)	<0.001
Eggs (g/day)	48.11 (52.66)	34.32 (40.18)	20.65 (32.46)	<0.001
Diet variety(g/day)	92.79 (41.66)	82.83(44.44)	59.08 (36.73)	<0.001
Cooking oil(g/day)	0.00 (0.00)	0.01 (0.30)	0.15 (3.05)	0.073
Salt	0.00 (0.00)	0.00 (0.02)	0.02 (0.58)	0.246
Alcohol (drinks/week)	1.54 (7.9)	1.6(9.4)	0.6 (2.8)	<0.001
drinking water (L/day)	873.86(592.12)	740.06(281.37)	539.42(215.71)	<0.001

All food subgroups are expressed as mean (SD).

### Diet quality distance trajectories and the risk of T2DM.

The association between diet quality distance trajectories and T2DM is represented in Table 4. During the follow-up period, 332 participants were identified as having T2DM. Compared to class 1, the multivariate-adjusted models showed an increased risk of T2DM in all classes for all models. In class 2 (HR = 3.81, 95% CI: 1.69-8.58); (HR = 4.14, 95% CI: 1.85-9.25) and (HR = 4.40 95%CI: 2.02-9.59) for models 1, 2, and 3 respectively. Class 3 (HR = 3.04, 95%CI: 1.77-5.23); (HR = 3.47, 95% CI: 1.96-6.12); and (HR = 3.68; (95% CI: 2.11-6.43) for models 1, 2, and 3 respectively. Using class 3 as the reference since it had the lower intake of meat products, after adjusting for all covariates, we still found an increased risk of T2DM in class 1 (HR = 3.28, 95% CI: 1.91-5.63, (HR = 2.88, 95%CI: 1.63-5.08) and (HR = 2.71, (95% CI: 1.55-4.73) for model 1,2, and 3 respectively. There was no significant association for class 2 considering class 3 as reference group.

**Table 4:** Hazard ratios for diabetes according to changes in diet quality distance.

**Trajectories**	**Model 1**	**Model 2**	**Model 3**
Diet Quality Distance (overall unbalance)			
Moderate to persistent decline (class1)	1	1	1
High to persistently moderate (class2)	3.81(1.69-8.58)	4.14(1.85- 9.25)	4.40(2.02- 9.59)
High stable(class3)	3.04(1.77-5.23)	3.47(1.96- 6.12)	3.68 (2.11-6.43)
Diet Quality Distance Score (overall unbalance)			
High stable(class3)	1	1	1
High to moderate(class2)	1.25(2.49-6.30)	1.19(2.26-6.29)	1.19(2.22- 6.44)
Moderate to persistent decline(class1)	3.28(1.91-5.63)	2.88(1.63-5.08)	2.71(1.55- 4.73)

Model 1: adjusted for age +gender +body max index + smoking status+ MET-h/week+ energy intake.

Model 2: model1 +urbanicity+ income+regions+area+ education level.

Model3: model2 + hypertension.

### Diet quality distance trajectories and T2DM biomarkers

Table 5 shows the T2DM biomarkers' differences across all trajectories. Fasting Plasma Glucose (FPG), HbA1c (hemoglobin), HDL-C, Uric acid, and triglyceride concentrations in class 2 and class 3 were higher than class 1 (all p for trend < 0.05). High-sensitivity C-reactive protein (hs-CRP) and HOMA-IR in all trajectories showed non-significant higher trends (*p* >0.05).

**Table 5:** Type 2 diabetes biomarkers differences across diet quality distance trajectories across Chinese adults.

**Biomarkers**	**DQD trajectories**			***P value***
	**Class1**	**Class2**	**Class3**	
HbA1C (%)	5.46(0.03)	5.47(0.15)	5.65 (0.05)	<0.001
FPG (mmol/l)	5.21(0.25)	5.29(0.14)	5.34(0.08)	<0.001
HOMAIR	2.31(1.73)	3.69 (0.97)	3.36 (0.55)	0.06
HDL-C (mmol/l)	1.26 (0.13)	1.38(0.07)	1.43 (0.04)	<0.001
UA (μmol/l)	285(20.74)	312(11.62)	303(6.61)	<0.001
hs-CRP (mg/l)	2.81 (3.52)	1.33(1.12)	1.24 (1.97)	0.44
TG (mmol/l)	1.14 (0.26)	1.56 (0.14)	1.48 (0.08)	<0.001

Data are mean (Standard Error). FPG, fasting plasma glucose; TG, triacylglycerol; UA, uric acid; hs-CRP, high-sensitivity C-reactive protein; HOMAIR, Homeostatic Model Assessment for Insulin Resistance.

### Association between diet quality distance trajectories and T2DM related markers.

We implemented a random-effects model to describe the change in biomarkers among the three classes (see Table 6). After adjusting for all potential covariates, no associations were observed across all trajectories with all identified biomarkers (*p* values > 0.05) (Table 6).

**Table 6:** HBA1C, FPG, HDL-C, HOMA-IR, UA, and TG changes across DBI_DQD trajectories.

**Biomarkers/Trajectories**	**Model 1**	**Model 2**	**Model 3**
HBA1C			
Class1	ref	ref	ref
Class2	2.54(0.97-1.02)	1.82(0.97-1.02)	1.64(2.41-2.74)
Class3	9.28(0.98-1.03)	1.37(0.99-1.03)	1.35(9.72-3.68)
FPG			
Class1	ref	ref	ref
Class2	4.95(-2.99-3.99)	5.41(-2.94-4.02)	6.63(-2.84-4.10)
Class3	2.81(-2.87-3.43)	4.76(-2.671- 3.62)	5.41(-2.59-3.67)
HDL-C			
Class1	ref	ref	ref
Class2	9.59(-5.09-7.01)	9.83(-5.07- 7.03)	1.06(-4.99-7.12)
Class3	2.00(-3.44-7.45)	1.88(-3.57-7.33)	1.94(-3.51-7.40)
HOMAIR			
Class1			
Class2	4.23(-1.15-1.99)	4.05(-1.17-1.98)	3.77(-1.20-1.96)
Class3	4.71(-9.45-1.88)	5.55(-8.67-1.97)	5.33(-8.92-1.95)
UA			
Class 1			
Class 2	3.78(-2.18-9.76)	3.61(-2.36-9.59)	3.52(-2.46-9.52)
Class3	2.74(-2.65-8.14)	2.35(-3.05-7.76)	2.27(-3.14-7.70)
TG			
Class1			
Class 2	1.03(-1.44-2.21)	1.03(-1.53-2.22)	9.98(-1.89-2.18)
Class 3	7.09(-3.58-1.77)	7.06(-3.67-1.78)	6.77(-3.97-1.75)

Model 1: adjusted for age +gender +bmi + smoking status + MET-h/week +energy Intake.

Model 2: model1 +urbanicity + income + regions + area+ education level.

Model 3: model2 + hypertension.

## DISCUSSION

This study sought to examine the association of long-term diet quality changes as assessed by the revised DBI-07 with T2DM and its biomarkers across 14 years among Chinese adults. We observed elevated diet quality distance levels among Chinese adults with a dominant insufficient intake of most food groups. We derived three different variations of diet quality across the 14 years follow-up. Three variants of diet quality represented as trajectories were derived from repeated DBI-DQD over 14 years. All trajectories had somewhat significant distinct clinical profiles and food group intake.

Participants tended to consume vegetables in adequate quantities across all trajectories while cooking oil, salt, alcohol, and water were consumed in low amounts. Other food groups were consumed in inadequate/excessive quantities. In addition to the similarities mentioned above across all three trajectories, the first diet pattern derived from diet quality distance was characterized by a diet high in meat products, cereals and low in fruits, eggs, dairy, soybean, diet variety, and adequate for fish and shrimps intake. This diet pattern was defined as the lower unhealthier group (class 1) corresponding to Chinese adults who decreased their DQD level from moderate to low. The second diet pattern was characterized by an adequate intake of meat products, fish, and shrimps; fruits, dairy, soybean, and diet variety intakes were insufficient. Defined as moderate unhealthier group (class 2), it corresponded to Chinese adults with a high DQD level at early adulthood, whose level persistently declined and remained moderate with age. Finally, the third diet pattern was characterized by participants with a diet low in meat products, fishery, eggs, fruit, dairy, soybean, and diet variety corresponding to Chinese adults with a persistently high DBI-DQD level (class 3) defined as the higher unhealthier group. Most participants had a high DBI-DQD level representing almost 92% of the studied population. All diet quality distance trajectories were associated with an increased risk of T2DM. There were no significant changes of T2DM biomarkers across all trajectories, although the second and third trajectory had higher circulating concentrations of identified biomarkers as compared to the first.

Salt and water intake were insufficient in all classes in our study. Most health guidelines recommend low salt intake, mainly based on correlations between high dietary sodium and increased blood pressure [23]. Nevertheless, studies in both humans and animals have shown that a diet too low in salt could increase insulin resistance and T2DM [24, 25]. Studies have suggested that the mechanism by which low salt intake could induce insulin resistance and lead to T2DM is similar to that in high sugar intake diets [26], while in other studies, law salt intake could induce insulin resistance by activating the renin angiotension aldosterone system [25]. A previous report by Evan C Johnson et al*.* has shown that in addition to low salt intake activating RAAS, low daily total water intake could also increase arginine vasopressin (AVP), both of which lead to T2DM [27, 28]. Vasopressin is usually elevated in healthy persons that habitually consume low volumes of water. Elevated vasopressin increases blood glucose through the hypothalamic system's stimulation, releasing a hormone prompting increased cortisol and eventually unwarranted cortisol-mediated gluconeogenesis [27, 28].

In our study, the Chinese adults clustered in the first trajectory had a high intake of red meat products, poultry, and game. Diets high in meat have been related to T2DM and other chronic diseases in several studies. Similar to our findings, Steinbrecher et al*.* [29] in their multiethnic cohort, where meat intake was assessed at baseline, after 14 years of follow-up time, meat intake was positively associated with T2DM [29]. Evidence from clinical, experimental, and epidemiological studies has established the harmful effects of meat products in the occurrence of T2DM and its comorbidities [30, 31]. Poor insulin regulation has been linked to the high consumption of meat products [30]. Since they are high in amino acids, fat, and proteins, meat products are usually fattier than legumes, vegetables, and some cereals. Fats in meat are generally saturated, and there are various mechanisms by which such fats could lead to T2DM [30, 32, 33]. Other studies established that high consumption of meat may contribute a load of dietary fat that enhances intracellular lipid storage and weakens insulin metabolism, which increases resistance [32]. The abundant fat and amino acids in meat products can also increase insulin response, which reduces fat oxidation [33]. In our findings, no significant changes in lipid markers were found across all trajectories. Inflammation has also been involved in high meat consumption and T2DM. The hs-CRP, a biomarker of inflammation, was found in elevated concentrations across all trajectories in our study. However, the high meat intake class had the highest level of Hs-CRP. In recent reports, high meat-based diets were associated with high levels of biomarkers of inflammation [34] and as meat intake increased, so did the biomarkers' level. Our findings are somewhat consistent with prior findings.

Participants who shared the diet pattern in the second trajectories tended to have adequate meat intake but higher cereals intake. In the Chinese dietary guideline, cereals encompass rice, potatoes, wheat noodles, bread, and buns known as a Chinese staple food. These foods are high in glycemic index and could impair cellular membranes' physical properties and the binding affinity of insulin receptors and promote hyperglycemia, inflammation, and increase oxidative stress [34], leading to T2DM. Consumed in adequate quantities, fish could reverse the mechanism mentioned above since it contains Omega-3 fatty acids, which are anti-inflammatory and could improve cellular membranes' physical properties and the binding affinity of insulin receptors, reducing the risk of T2DM [35]. Eggs intake was inadequate across all classes in our study. Although eggs have various nutrients that could improve cardio-metabolic functions, the eggs-T2DM relationship is inconsistent. While previous studies reported no adverse changes in cardiovascular risk markers between eggs and T2DM and that high egg intake could raise the risk of T2DM by 13% [36], recent ones suggest that high egg consumption is associated with a lower risk of T2DM [37].

Red meat products, poultry, and game intake were consumed in low amounts across the third trajectory, while vegetables, cereals were consumed in sufficient quantities but with a high risk of T2DM. Our findings contradict the beneficial effect of a low-meat diet effect on T2DM status as established by Kitada et al. [38], who demonstrated that low protein diets could improve glucose homeostasis. In mice, low protein diets prevented dysregulation of glucose levels by inducing liver stress signaling pathways [38]. Our findings are consistent with a review by Barnard et al., who found that even a small intake of meat products increased the risk of developing T2DM among long-term non-vegetarians [39]. More so, Vang et al. found that after 17 years of follow-up, subjects who consumed meat once per week still had a high risk of developing T2DM [31], and later on, meat was suggested to be added to the T2DM list of risk factors [30]. Overall, these findings indicate that the risk related to meat intake and /or cereals, salt intake, and water intake in our study may be due to the insufficient intake of certain food groups contributing to a preventive profile for T2DM. These food groups that have been identified as protective against T2DM by Chinese diet guidelines, food pagoda, and other health guidelines include: fruits, soybean, dairy, whole grains, nuts, and diet variety [23]. Rich in fibre, antioxidants, phytochemicals, lysine, leucine, isoleucine, phenylalanine, calcium, and phosphate, these food groups are known to increase glycemic control and insulin sensitivity if consumed adequately. Various dietary intervention trial data indicated that if consumed in adequate quantities, these food groups can reduce the blood concentration of inflammatory markers and reduce the risk of T2DM [40, 41]. Across all the derived trajectories, these food groups were consumed in insufficient quantities with an increased risk of T2DM. Individuals in the second trajectory had the highest risk of T2DM. In addition to diet-related factors prone to T2DM, other factors such as age, blood pressure, and hypertension contribute to cardiovascular risk, which is the principal risk in T2DM [42]. Individuals across class 2 were older, had a higher mean of blood pressure, and a higher prevalence of hypertension, which could also explain its highest risk of T2DM compared to other classes. Considering the increased risk of T2DM in all the studied trajectories, our findings indicate that a long-term unhealthy/imballanced diet characterized by high DQD level is significantly associated with an increased risk of T2DM. Gradually reducing DQD level from moderate to low or from high to moderate has not made a difference in being at risk of developing T2DM or not. However, this cannot be said for T2DM biomarkers changes across trajectories since no significant change in T2DM biomarkers was found across all the trajectories.

Our study has important strengths. First, to our knowledge, this is the first study to classify diet quality as assessed by the revised DBI-07 in a Chinese population into trajectory classes and explore the association between diet quality distance trajectories and T2DM risk and the changes in T2DM related biomarkers. Previous studies used baseline dietary quality indices as the relevant exposure, ignoring potential diet fluctuations between time points during follow-up. Ignoring these fluctuations could obscure the effect of changes in dietary patterns throughout adult life course. In the present study, we used an innovative method called latent class mixed models to classify the diet quality distance sharing similar dietary quality distance levels. Second is the steadiness and the consistency of the dietary intakes over the period of CHNS and the 14 years follow-up of this study.

Our study also has some limitations. This study included only Asian participants, which could limit the generalizability of our findings to other populations. T2DM ascertainment was mainly based on self-report since only a one-time point of T2DM related biomarkers in the 2009 CHNS was available, which could have biased the linear regression results.

## CONCLUSIONS

Our findings highlight the damaging effects of long-term unhealthy diet characterized by high diet quality distance levels on the future risk of T2DM. Gradually reducing diet quality distance to moderate to low level may not make a difference, which shows the importance of adopting healthy eating habits at early adulthood. From a public health standpoint, considering the implications of our findings, there is a vital need for approaches promoting the adoption of healthy eating habits at early adulthood as recommended by the Chinese resident dietary guideline to prevent the risk of developing T2DM among Chinese adults. Chinese dietary guideline recommends eating a variety of foods in a cereal-based diet. Eat plenty of vegetables, fruits, dairy products, and soybeans, eat moderate amounts of fish, poultry, eggs, meat, limit salt, cooking oil, alcohol, and drink enough water to prevent cardio-metabolic diseases and other chronic diseases [23]. Further studies on a non-Asiatic population are needed to validate our findings.

## MATERIALS AND METHODS

### Ethics statement

The China Health and Nutrition Survey (CHNS) used for this study was approved by the Institutional Review Committees of the University of North Carolina at Chapel Hill; the China-Japan Friendship Hospital, the Chinese Ministry of Health Institute of Nutrition and Food Safety, and China Centers for Disease Control. The subjects gave informed consent for participation.

### Study design and population

This study used the data collected by the CHNS, which is an open, prospective survey designed to examine the association between economic and social changes and a range of health behaviours across space and time [43]. The CHNS collected health data in 228 communities throughout China from 1989 to 2011 in eight survey rounds. A multistage, random cluster sampling method was used to select the samples from nine provinces (Liaoning, Heilongjiang, Jiangsu, Shandong, Henan, Hubei, Hunan, Guangxi, and Guizhou) in China. Questionnaires were used to collect the following data: social and economic factors, health factors, nutrition factors, and population factors of sociology. Blood samples were collected only for the first time in 2009. More details about the CHNS data have been described elsewhere [44].

### Selection criteria

### Exclusion criteria

A total of 25611 adults (men and women) provided a 3-day 24h dietary recall from 1997-2011.

Were excluded from this study subjects,

• less than 18 years old (n = 10415);

• more than 65 years old (n = 891);

• with extreme values of daily energy intake less than 800 and more than 4200 for men (n =225), energy intake less than 500 and more than 3500 for women (n = 281),

• with prior diagnosis of obesity (n = 542), hypertension (n = 2822), diabetes (n = 189), other cardiovascular diseases (n=14) at wave of entry;

• pregnant women (n= 181);

• with only one-year survey (n=2104)

### Inclusion criteria

**Figure 2:**
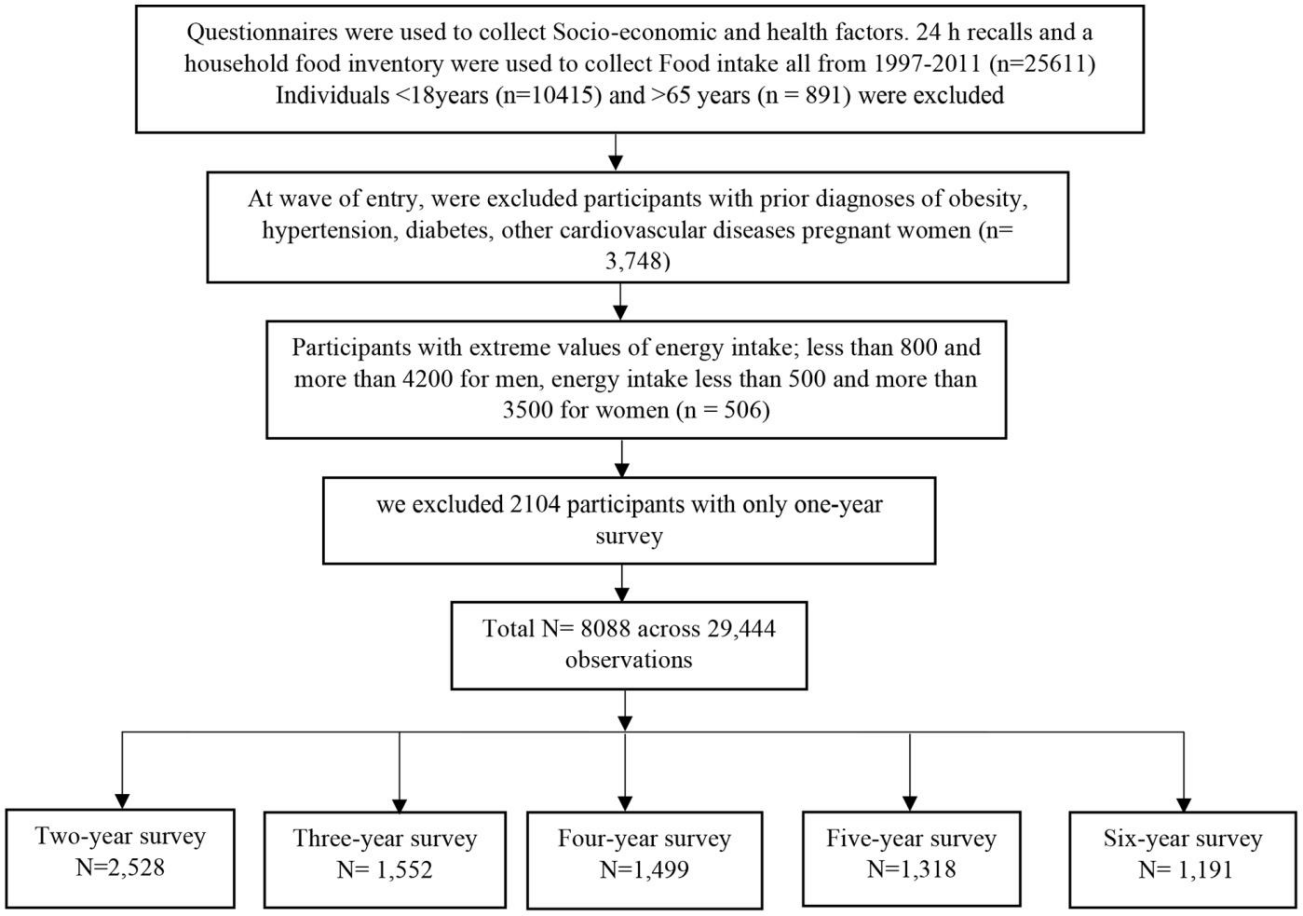
**Flow chart of participants' selection.**

A total of 8088 participants aged from 18-65 years old, without underlying conditions, unpregnant, within acceptable daily energy intake limits, with more than one-year survey, were included in this study. The number of visits ranged from two to six surveys (two visits, n = 2,528; three visits, n = 1,552; four visits, n =1,499; five visits, n = 1,318; six visits, n = 1,191; median = four visits; across 29,444 observations). Figure 2 is a flow chart showing participants' selection.

### Dietary assessment and food grouping

The food intake was measured over three consecutive days (including two weekdays and one weekend day) with 24 h recalls and a household food inventory. Every household member aged two and above was asked to report any food consumed, whether at home or away from home. Trained interviewers recorded the types and amounts of food consumed at each meal via a face-to-face interview with food image and model aids. During the past 12 months, participants were asked about the drinking frequency and the number of alcoholic beverages (such as high alcohol liquor, low alcohol liquor, wine, beer, etc.). Alcohol was converted to DBI-07 criteria in each alcoholic beverage [45] and then summed up to calculate average alcohol consumption per day. After carefully reviewing the dietary data, food group classifications were summed up corresponding to the Chinese Table of Food Composition [46].

### Dietary balance index-07

The revised Chinese DBI-07 (revised from the DBI-2002) aims to assess the Chinese population's overall dietary quality. The DBI-07 is based on the core items of the Chinese dietary guide [23] formulated by the Chinese society of nutrition. The core items are composed of seven components, including (1) cereals; (2) vegetables and fruits; (3) milk and dairy products, soybean and soybean products; (4) Animal food; (5) condiments and alcoholic beverage; (6) Diet variety; (7) drinking water [45]. For each component of DBI-07, a score of 0 indicates that the recommended intake amounts are met. In DBI-07, positive scores (range= 0–12) are used to assess the excessive intake levels of alcoholic beverages and condiments, which are recommended to be consumed in a "reduced" or "limited" amount according to the guidelines. The DBI-07 gives negative scores (range = -12–0) assessing inadequate intake of vegetables and fruits; dairy products, soybean and soybean products; dietary variety and drinking water recommended by nutritional guidelines to be consumed 'plenty' or 'sufficient' amounts. Furthermore, DBI-07 also used both negative and positive scores for cereals (range = -12–12) and animal food (range = -12–8), which the dietary guidelines recommended consuming in 'appropriate amount'. The scoring of the 7 components is based on seven levels of energy intake when considering the difference in nutrient requirements in energy consumption. To evaluate the diet variety in DBI-07,12 identified food subgroups are used: 1) rice and rice products; 2) wheat and wheat products; 3) corn, coarse grains, and products, starchy roots and products; 4) dark-colored vegetables; 5) light-colored vegetables; 6) fruits;7) soybean and soybean products; 8) milk and dairy products; 9) red meat and meat products; 10) poultry and games; 11) eggs; 12) fish and shellfish. A food subgroup was assigned a score of 0 if the intake reached or exceeded the lowest recommended intake else; a score of −1 was assigned. For soybean and products, the lowest recommended intake is 5 g, and for the other 11 food subgroups, 25 g. Diet variety component scores vary between −12 to 0. There are diet quality indicators calculated based on the scores for each DBI-07 component. Higher bound score (HBS), the excessive food intake indicator, is calculated by adding all the positive scores. Lower bound score (LBS), the indicator of inadequate food intake, is calculated by adding all the negative scores. Diet quality distance (DQD) assesses imbalanced food intake by adding absolute values of both positive and negative scores. If the overall score is negative, it shows that the average intake of food is insufficient; if the overall score is positive, it shows that the average intake of food is excessive. [45, 47, 48] More details on DBI-07 can be found in “The Chinese Diet Balance Index revised” [45].

### Outcome ascertainment and anthropometric variables assessment T2DM ascertainment

T2DM was identified by self-reports of a history of T2DM diagnosis and/or fasting blood glucose ≥7.0 mmol/l and/or HbA1c ≥40 mmol/mol (6.5%), and/or receiving diabetes treatment. Detailed information on the treatment for T2DM included six sections: (1) special diet; (2) weight control; (3) oral medication; (4) insulin injection; (5) Chinese traditional medicine; (6) home remedies. Data for the incidence of self-reported T2DM were first collected in 1997, and subsequently in 2000, 2004, 2006, 2009 and 2011. Fasting blood glucose and HbA1c were also used to identify incident diabetes cases in the 2009 survey. The total incident T2DM included was 332.

### Body mass index

Weight and height were measured in light clothing and without shoes. Weight was measured to the nearest 0.1 kg using a calibrated scale; and height to the nearest 0.2 cm using a portable stadiometer. BMI was calculated by dividing body weight by height in meters squared.

### Hypertension

Elevated blood pressure was defined as an average of systolic blood pressure ≥140mmHg, and /or an average of diastolic blood pressure ≥90 mmHg. Participants were classified as having hypertension if their systolic blood pressure and/or diastolic blood pressure or both had exceeded the values above if they were taking hypertension drugs, and/or if they were diagnosed with high blood pressure.

### Other variables assessment

Demographic characteristics were collected by trained interviewers using standard questionnaires for each individual at-home visits, including age, gender, area of residence, household income, highest education level. Based on population density, the area of residence was categorized as urban or rural. Physical activity level (PAL) was measured using questionnaires that collected the time spent in all activities in a typical week, including work, leisure, travel, and household chores. A summary measure expressed as total metabolic equivalents (METs) was created by multiplying the time spent in each activity with the METs of each activity. The time spent in each activity was converted into metabolic equivalent of task (MET) hours per week based on the compilation of physical activities [49]. The Urbanicity index was defined using 12 multidimensional components reflecting the heterogeneity in economic, social, demographic, and infrastructural changes at the community level. Further, we also considered geographical factors as northern, East Coast, and central provinces. Smoking status and total energy intake were also considered as potential confounders.

### Statistical analysis

LCMM was used to derive 14 years of DBI-DQD trajectories using the revised DBI-07. DBI-DQD is an indicator of the Diet Balance Index for Chinese derived from adding the absolute value of both DBI-LBS and DBI-HBS as described above. DBI-DQD informs us about the level of imbalance of one's diet.

LCMM clustered Chinese adults according to the similarities in DBI-DQD. The membership to class trajectories was treated as the exposure to evaluate the association with T2DM. We identified 3 classes of subjects with comparable DQD throughout 14 years of diet intake. The survey age was used as a timescale for the trajectories. We modelled the DBI-DQD trajectories among participants who were recruited in all wave. To determine the best fit of our models, we used rigorous criteria such as the lowest Bayesian information criterion, including at least 2% of the sample population within each trajectory class. Once DBI-DQD trajectories were determined and all individuals assigned to corresponding trajectories, a chi-square test was used to assess the differences for categorical variables and ANOVA to assess the differences for continuous variables. Food components were also compared among the trajectories to determine their differences in food intake.

Furthermore, we conducted a multivariate mixed-effect cox regression model clustered by ID (identifier) with Breslow method to account for time-varying covariates. Hazard ratio (HR) was used to estimate the association between three DBI-DQD trajectories and T2DM incidence. A random effect linear regression was also used to examine the changes in T2DM biomarkers. A two-sided *p* values < 0.05 was considered statistically significant. DBI-DQD and T2DM biomarkers were log-transformed to improve the normality of the distribution. All statistical analyses were performed using R 3.5.3 (https://www.r-project.org/).

### Data availability

The Data from China Health and Nutrition Survey used in this study can be downloaded at https://www.cpc.unc.edu/projects/china.

## Supplementary Material

Supplementary Table 1
