# Supplementary material for: Association of fourteen years diet quality trajectories and type 2 diabetes mellitus with related biomarkers
Source: Aging (Albany NY). 2021 Mar 26;13(7):10112–27. doi: 10.18632/aging.202771 (PMC8064195; doi:10.18632/aging.202771)
Supplement: Supplementary Table 1 [file aging-13-202771-s001.docx]

**Supplementary Table 1. Distribution of DBI-07 food subgroups scores by DQD trajectories (%).**

| Score | Cereals | | | Vegetables | | | Fruits | | | Dairy | | | Soybean | | | Drinking Water | | | Red Meat Products Poultry and Game | | | |
| --- | --- | --- | --- | --- | --- | --- | --- | --- | --- | --- | --- | --- | --- | --- | --- | --- | --- | --- | --- | --- | --- | --- |
|  | Class1 | Class2 | Class3 | Class1 | Class2 | Class3 | Class1 | Class2 | Class3 | Class1 | Class2 | Class3 | Class1 | Class2 | Class3 | Class1 | Class2 | Class3 | Class1 | Class2 | Class3 |  |
| (-12) -(-11) | 6.9 | 13.9 | 13.0 |  |  |  |  |  |  |  |  |  |  |  |  | 0.4 | 0.2 | 2.1 |  |  |  |  |
| (-10) -(-9) | 0.8 | 3.0 | 2.9 |  |  |  |  |  |  |  |  |  |  |  |  | 2.8 | 7.2 | 23.6 |  |  |  |  |
| (-8)– (-7) | 3.1 | 4.3 | 4.4 |  |  |  |  |  |  |  |  |  |  |  |  | 13.3 | 24 | 41.7 |  |  |  |  |
| (-6)- (-5) | 3.1 | 1.9 | 2.4 | 0.1 | 0.4 | 0.3 | 42.7 | 63.5 | 81.1 | 73.1 | 89.6 | 97.8 | 21.2 | 28.7 | 48.3 | 29.9 | 31.9 | 22.6 |  |  |  |  |
| (-4)- (-3) | 1.5 | 2.7 | 1.5 | 20.7 | 34.5 | 35.4 | 24.4 | 20.9 | 12.1 | 10.7 | 4 | 1 | 7.1 | 10.7 | 8.8 | 26.9 | 23.2 | 7.1 | 1.6 | 23.9 | 34.5 |  |
| (-2)- (-1) | 14.5 | 6.0 | 3.0 | 35.1 | 30.9 | 28 | 24.4 | 11.4 | 4.9 | 13.5 | 5 | 1.1 | 18.2 | 10.1 | 8.8 | 16.4 | 8.7 | 2 | 17.2 | 13.4 | 19.7 |  |
| 0-1 | 16 | 12.8 | 17.2 | 44.1 | 34.1 | 36.3 | 8.5 | 4.2 | 1.9 | 2.8 | 1.4 | 0.1 | 53.5 | 50.4 | 34.2 | 10.3 | 4.8 | 0.9 | 25.0 | 25.7 | 23.7 |  |
| 2-3 | 17.6 | 14.6 | 5.3 |  |  |  |  |  |  |  |  |  |  |  |  |  |  |  | 26.6 | 20.9 | 14.6 |  |
| 4-5 | 16 | 11.9 | 10.5 |  |  |  |  |  |  |  |  |  |  |  |  |  |  |  | 29.7 | 16.1 | 7.5 |  |
| 6-7 | 8.4 | 9.0 | 9.2 |  |  |  |  |  |  |  |  |  |  |  |  |  |  |  |  |  |  |  |
| 8-9 | 6.9 | 6.7 | 10.4 |  |  |  |  |  |  |  |  |  |  |  |  |  |  |  |  |  |  |  |
| 10 -11 | 2.3 | 4.7 | 7.2 |  |  |  |  |  |  |  |  |  |  |  |  |  |  |  |  |  |  |  |
| 12 | 3.1 | 8.6 | 13 |  |  |  |  |  |  |  |  |  |  |  |  |  |  |  |  |  |  |  |

| Score | Fish and shrimp | | | | Eggs | | | Cooking oil | | | Salt | | | Alcohol | | | Diet variety | | |
| --- | --- | --- | --- | --- | --- | --- | --- | --- | --- | --- | --- | --- | --- | --- | --- | --- | --- | --- | --- |
|  | Class1 | Class2 | Class3 | | Class1 | class2 | Class3 | Class1 | Class2 | Class3 | Class1 | Class2 | Class3 | Class1 | Class2 | Class3 | Class1 | Class2 | Class3 |
| (-12) -(-11) |  |  | |  |  |  |  |  |  |  |  |  |  |  |  |  | 9.1 | 13.8 | 19.3 |
| (-10) -(-9) |  |  | |  |  |  |  |  |  |  |  |  |  |  |  |  | 0.2 | 1.5 | 10.3 |
| (-8)– (-7) |  |  | |  |  |  |  |  |  |  |  |  |  |  |  |  | 7 | 16.5 | 30.6 |
| (-6)- (-5) |  |  | |  |  |  |  |  |  |  |  |  |  |  |  |  | 32.5 | 37.6 | 29.8 |
| (-4)- (-3) | 30.6 | 39.4 | | 75.8 | 6.3 | 22.3 | 45.5 |  |  |  |  |  |  |  |  |  | 38.6 | 25.3 | 9.3 |
| (-2)- (-1) | 18.5 | 21.4 | | 8.6 | 12.2 | 17.0 | 19.9 |  |  |  |  |  |  |  |  |  | 12.6 | 4.9 | 0.7 |
| 0-1 | 50.9 | 39.2 | | 15.6 | 16.3 | 21.1 | 19.2 | 100 | 100 | 99.8 | 100 | 100 | 99.8 | 97.7 | 97.7 | 100 | 0 | 0.4 | 0 |
| 2-3 |  |  | |  | 10 | 8.9 | 7.2 |  |  | 0.1 |  |  | 0.1 | 1.2 | 1.0 |  |  |  |  |
| 4-5 |  |  | |  | 55.2 | 30.7 | 9 |  |  | 0.1 |  |  | 0.1 | 1 | 1.2 |  |  |  |  |
| 6-7 |  |  | |  |  |  |  |  |  |  |  |  |  |  |  |  |  |  |  |
| 8-9 |  |  | |  |  |  |  |  |  |  |  |  |  |  |  |  |  |  |  |
| 10-11 |  |  | |  |  |  |  |  |  |  |  |  |  |  |  |  |  |  |  |
| 12 |  |  | |  |  |  |  |  |  |  |  |  |  |  |  |  |  |  |  |
